# A novel nitrogen heterocycle platform-based highly selective and sensitive fluorescence chemosensor for the detection of Al^3+^ and its application in cell imaging†

**DOI:** 10.1039/c8ra10036e

**Published:** 2019-02-12

**Authors:** Zengchen Liu, Shujing Li, Genwu Ge, Yanxia Li, Chunxiang Zhao, Hui Zhang, Zhiguang Yang

**Affiliations:** College of Chemistry and Chemical Engineering, Henan Key Laboratory of Rare Earth Functional Materials, International Joint Research Laboratory for Biomedical Nanomaterials of Henan, The Key Laboratory of Rare Earth Functional Materials and Applications, Zhoukou Normal University Zhoukou 466001 PR China liuzengchen@zknu.cn hxzhanghui2005@163.com

## Abstract

In the study, a highly selective and sensitive fluorescence sensor derived from nitrogen heterocycle was synthesized and characterized. By the fluorescence experiments, it was found to show a higher response toward Al^3+^ than other commonly coexistent metal ions in C_2_H_5_OH/H_2_O media (pH = 7.2). Moreover, the large binding constant (3.44 × 10^14^ M^−1^) between Al^3+^ and the sensor was calculated by fluorescence titration experiment. In addition, the synergistic effect mechanism due to photoinduced electron transfer (PET) and C=N isomerization was deduced according to the fluorescence behavior. In addition, the fluorescence imaging in living cells was studied systemically, which exhibited high fluorescence sensing activity toward Al^3+^.

## Abstract 2

In the study, a highly selective and sensitive fluorescence sensor derived from nitrogen heterocycle was synthesized and characterized.

## Graphical abstract

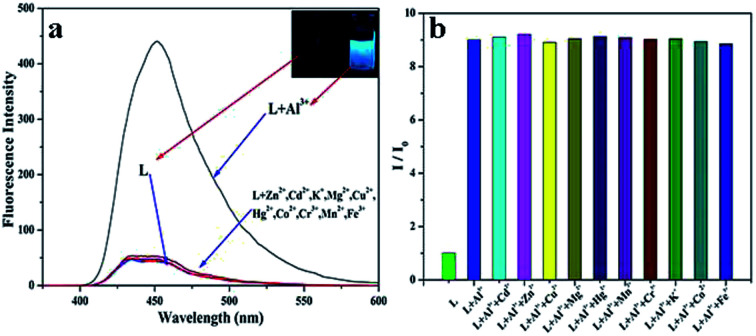

## Introduction

Recently, the highly selective and sensitive fluorescence chemsensors for detecting metal ions have attracted significant attention.^1–7^ This is because some relevant metal elements such as copper, zinc, mercury, chromium, and aluminium play very important roles in different organisms and the environment, which are closely related to some health and pollution problems. Thus, the monitoring of metal ions is of great significance. Among these common metals, aluminium is a non-essential metal element for bio-organisms. According to the reports of the World Health Organization, the average daily human intake of aluminium is approximately 3–10 mg.^8^ At present, aluminium is a proven neurotoxin, and the abnormal content of aluminium can cause many health hazards such as osteomalacia, Alzheimer's disease, and breast cancer. It can also damage the brain and kidneys.^9–11^ In addition, beyond the permissible concentration criterion of aluminium, the environment is adversely affected because it can enter the living organisms through the biosphere to induce the death of plants, soil pollution, and even diseases in humans.^12–17^ Due to the inadequate understanding of aluminium, it remains an important research topic. Thus, there is a considerable need for designing highly selective fluorescence sensors, which can dynamically and in real-time detect and rapidly analyze the aluminium element in biological and environmental system. In the living organism's environment, aluminium is mainly in the form of ions. Its ionic form also widely exists in the natural environment. Moreover, aluminium ion mainly exists in the aqueous system under physiological pH value condition. Therefore, it is a significant task to design the fluorescence sensor for Al^3+^ in aqueous media. To date, some fluorescence sensors for aluminium ion derived from organic molecules have been designed and reported.^18–21^ Consequently, considerable efforts need to be devoted for developing fluorescence sensors for Al^3+^ in aqueous media.^22,23^

When aiming at the rational development of highly selective fluorescence sensors for targeting aluminium ion, the choice of binding molecules is the dominant factor because in a complicated system many metal ions such as Fe^3+^, Co^2+^, and Cu^2+^ can interfere with the aluminium binding.^24,25^ Thus, it is very important to construct sensitive fluorescence chemosensors for aluminium ion in aqueous media. In continuation of our research topic based on metal ion fluorescence sensors,^26,27^ in the present investigation, a novel nitrogen heterocycle is developed to increase the selectivity and sensitivity for detecting Al^3+^. According to the spectral analysis, the fluorescence sensor exhibited high sensing property for Al^3+^ over other metal ions in the aqueous media (pH = 7.2). Compared with some reported fluorescence sensors for Al^3+^, the sensor derived from the heterocyclic compound exhibited unusual double ion coordination structures. Also, in the probe there are two highly efficient fluorophores, which are helpful in enhancing the luminescence efficiency. Moreover, the sensing mechanism was deduced to be due to the synergistic effect of photoinduced electron transfer (PET) and C=N isomerization, which arose from the coordination interaction between Al^3+^ and the sensor.

## Results and discussion

### Fluorescence selectivity and competition experiments

**Fig. 1:**
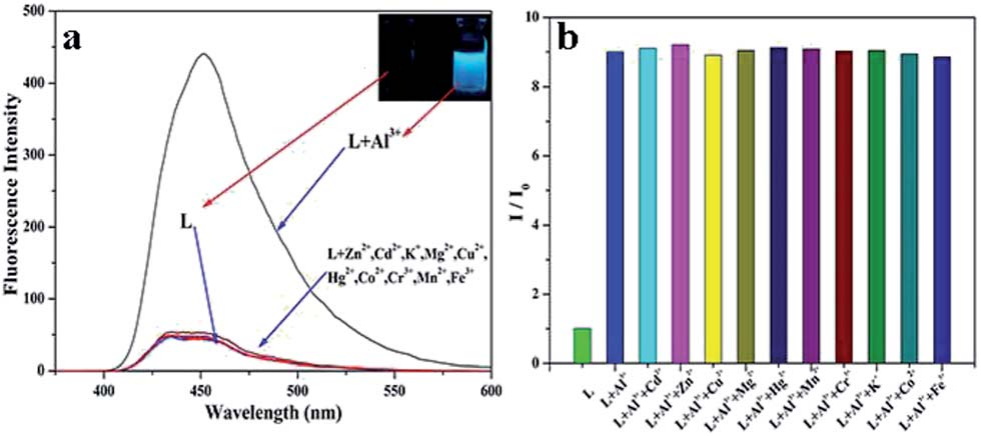
(a) The fluorescence selectivity spectrum of L (5 × 10^−6^ M) with various metal ions (5 × 10^−5^ M), inset: the fluorescence images of L and L + Al^3+^ in C_2_H_5_OH/H_2_O media under UV light, (b) the fluorescence competitive experiments with other cations.

The fluorescence selectivity activities toward various metal ions (Al^3+^, Cu^2+^, Zn^2+^, Cd^2+^, Hg^2+^, Mg^2+^, K^+^, Mn^2+^, Co^2+^, Cr^3+^, Fe^3+^) were investigated in C_2_H_5_OH/H_2_O (4 : 1) solution. As shown in Fig. 1a, upon addition of various metal ions, only Al^3+^ lead to the significant blue fluorescence signal. Except for the fluorescence increase for Al^3+^, other metal ions could not cause obvious fluorescence changes in L. It proved that the interaction between Al^3+^ and L gave rise to the large enhancement of fluorescence. The fluorescence selectivity behavior primarily indicated that L could act as a fluorescence probe for Al^3+^ under C_2_H_5_OH/H_2_O environment and physiological pH. This aqueous condition was an obvious advantage of the sensor. Moreover, to illustrate the high selectivity of L toward Al^3+^, the fluorescence images were also taken (Fig. 1a inset). The sensor L exhibited no fluorescence under UV light. Upon the addition of Al^3+^, the solution showed a remarkable light blue fluorescence, which indicated that L could act as a highly selective fluorescence sensor for Al^3+^ in C_2_H_5_OH/H_2_O media.

The high selectivity of L toward Al^3+^ was also investigated by the fluorescence competitive experiments with other cations. 10 equivalent Al^3+^ was added to the C_2_H_5_OH/H_2_O solution of L, then equivalent amount of other metal ions were also added into the same solution. The change in the fluorescence intensities was recorded. The histogram of the fluorescence changes is shown in Fig. 1b. As shown in the histogram, with addition of other metal ions, no significant variation in the fluorescence emission was observed by comparison with the fluorescence of L + Al^3+^. All the results indicated the high fluorescence selectivity of L towards Al^3+^ over other coexistent metal ions.

### Fluorescence titration and UV-Vis spectrum investigation

**Fig. 2:**
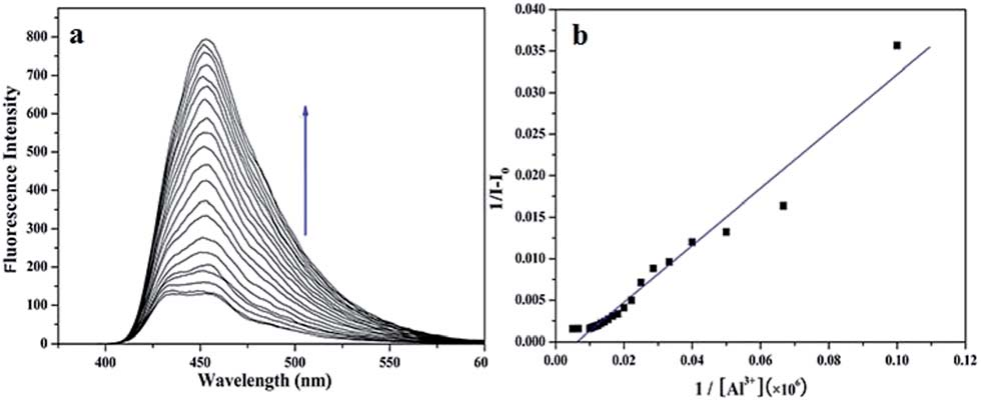
(a) Fluorescence titration spectrum of L (5 × 10^−6^ M) upon addition of 10 μL Al^3+^ (1 × 10^−3^ M) in C_2_H_5_OH/H_2_O (4 : 1) solution (pH = 7.2). Excitation at 350 nm, (b) fitting of fluorescence titration curve of L (pH = 7.2), *R* = 0.98243, SD = 0.00153.

Fluorescence titration experiment (Fig. 2a) of the sensor with Al^3+^ was performed at room temperature. Upon the addition of Al^3+^, the fluorescence signal at 450 nm was significantly enhanced. It explicitly proved that the binding between the sensor and Al^3+^ induced the change in the fluorescence of the sensor, which was in concurrence with the increase of the fluorescence intensity. In addition, as shown in Fig. 2b, the association constant between the sensor and Al^3+^ was estimated to be 3.44 × 10^14^ M^−1^ by fitting the data to the Benesi–Hildebrand expression with a good linear relationship, which demonstrated the high binding affinity between sensor and Al^3+^.

**Fig. 3:**
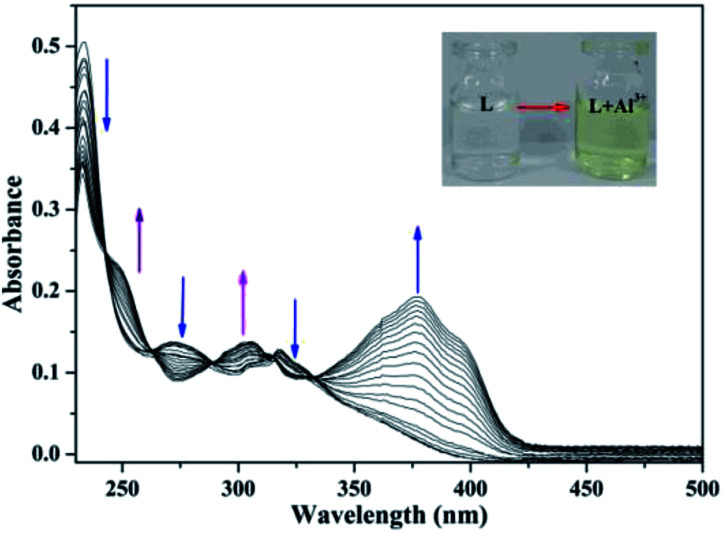
The UV-Vis absorption spectra of L (1.0 × 10^−5^ M) in C_2_H_5_OH/H_2_O (4 : 1) media in the presence of different amounts of Al^3+^ (0–4 equiv). The color change of the L solution after addition of Al^3+^ (inset).

The UV-Vis absorption spectrum of the sensor and Al^3+^ (pH = 7.2) was shown in Fig. 3, which exhibited the characteristic absorption of molecule L in the range 200–330 nm. With the addition of Al^3+^, the intensity of the absorption peaks at 230 nm, 275 nm and 320 nm decreased significantly. Simultaneously, the absorption intensity at 250 nm, 300 nm and 375 nm increased. The characteristic changes in the peaks (red shift) clearly indicated the coordination interaction between L and Al^3+^. This is because the coordination between the sensor and Al^3+^ lead to the energy increase of the n–π* transition from L, which resulted in the change in the UV-Vis spectrum. In addition, the new absorption band at 375 nm corresponded to the pale yellow color of the L–Al^3+^ solution (Fig. 3 inset).

### Fluorescence emission mechanism and detection limit

**Fig. 4:**
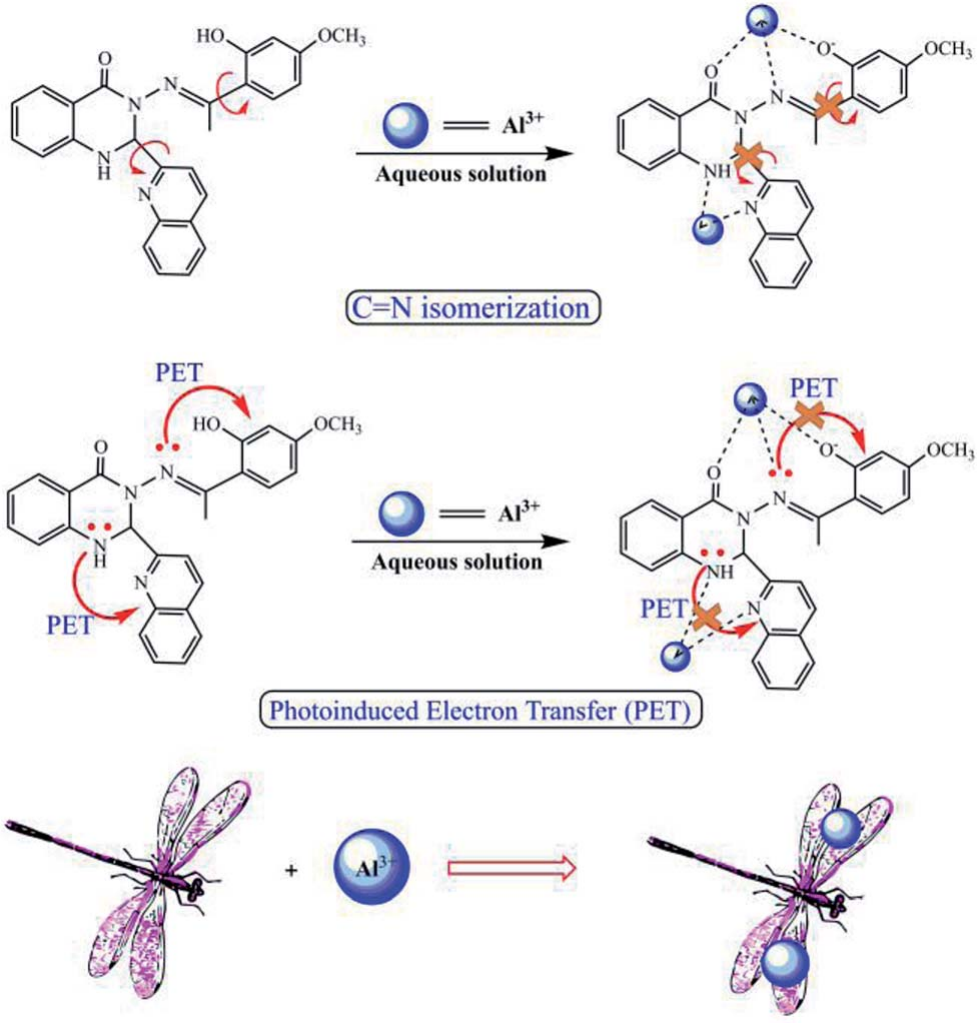
The deductive mechanism and fluorescence sensing process by L for Al^3+^ using fluorescence spectroscopy.

In accordance with the reported coordination sites, the fluorescence sensor was most likely to chelate with Al^3+^*via* its hydroxyl O, imino N, and N atoms from L. The predicted coordination mode is shown in Fig. 4. In addition, the supposed fluorescence mechanism was proposed (Fig. 4), which is ascribed to the synergistic effect due to photoinduced electron transfer (PET) and C=N isomerization processes between L and Al^3+^. Before L coordinated with Al^3+^, the solution of L exhibited no fluorescence. With the addition of Al^3+^, the free rotation of the benzene ring of paeonol and quinoline ring was restained, which was accompanied by the enhancement of the planarity and appearance of fluorescence. To evaluate the sensitivity of the sensor for Al^3+^, the detection limit of the fluorescence sensor for recognizing Al^3+^ was also tested through fluorescence spectroscopy. The fluorescence titration experiment of L with Al^3+^ demonstrated that the detection of Al^3+^ was at the magnitude level of 0.01 ppm, which is suitable for detecting Al^3+^ in living organisms and environmental samples.

### NMR and MS titration experiments

**Fig. 5:**
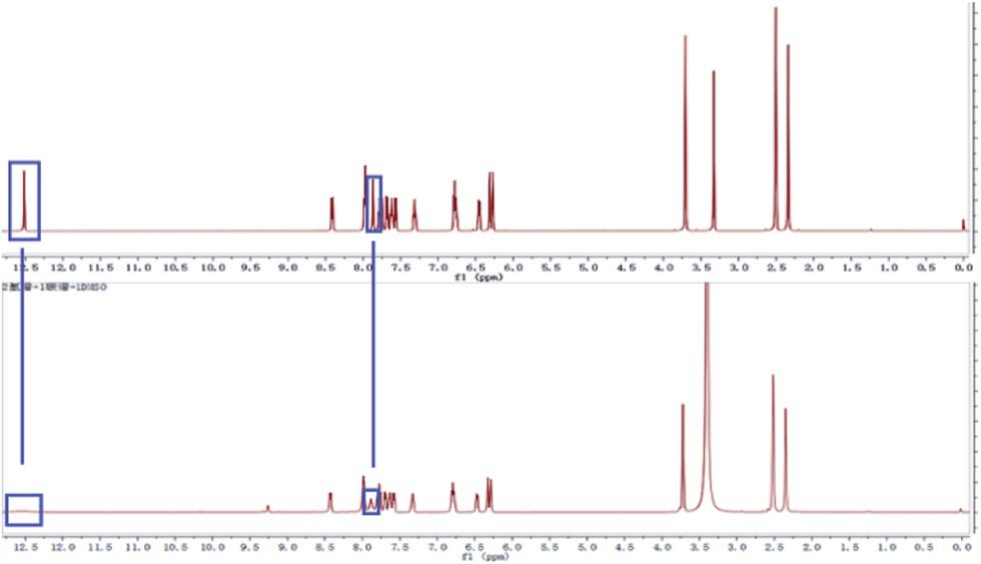
The NMR titration spectrum of L with Al^3+^.

**Fig. 6:**
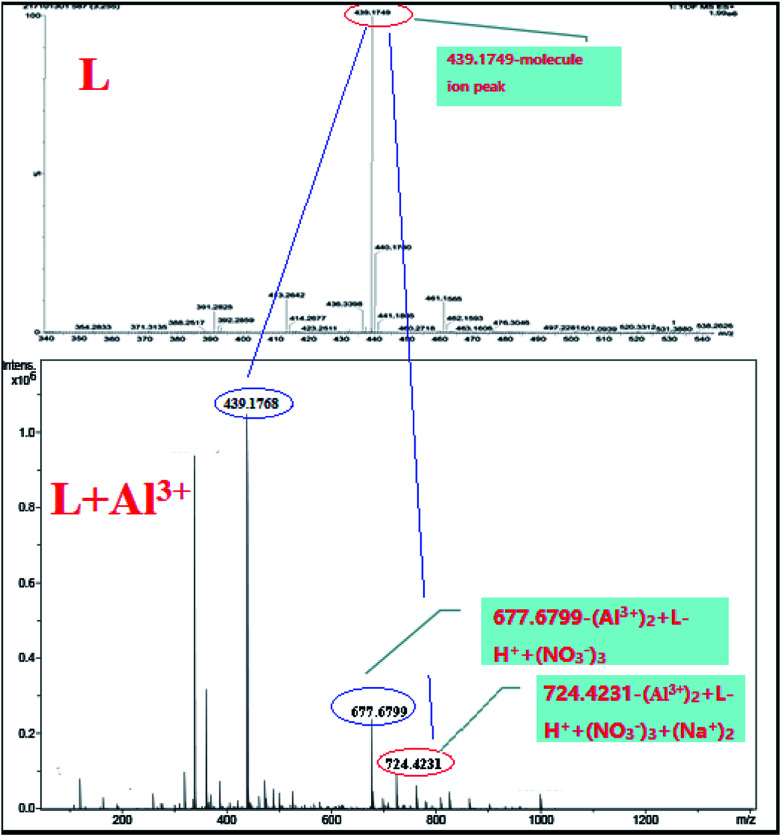
The HRESI-MS titration spectrum of L with Al^3+^.

To confirm the coordination ratio between L and Al^3+^, the NMR and MS titrations were tested systemically. According to the ^1^H NMR titration of the sensor with Al^3+^ (Fig. 5), the chemical shift (*δ* = 12.52) due to the reactive hydrogen of the hydroxide radical disappeared. In addition, the chemical shift (*δ* = 7.87) due to the reactive hydrogen of the imino group saw a marked change with a decreasing trend. These changes in the NMR signals indicated that the two groups (–OH and –NH–) could be acting as the coordination sites for Al^3+^. Also, in the process of coordination, the reactive hydrogen atom from the –OH is released. Moreover, the coordination modes were demonstrated by the HRESI-MS titration (Fig. 6). The MS peak at 439.1749 was the molecular ion peak of L. After the addition of Al^3+^, the new MS peaks at 677.6799 ((Al^3+^)_2_ + L–H^+^ + (NO_3_^−^)_3_) and 724.4231 ((Al^3+^)_2_ + L–H^+^ + (NO_3_^−^)_3_ + 2Na^+^) were accompanied by the generation of a new metal complex molecule, which emerged from the 1 : 2 coordination mode between L and Al^3+^.

### Cell fluorescence microscopy, cytotoxicity, and simple filter paper chromatography analysis

**Fig. 7:**
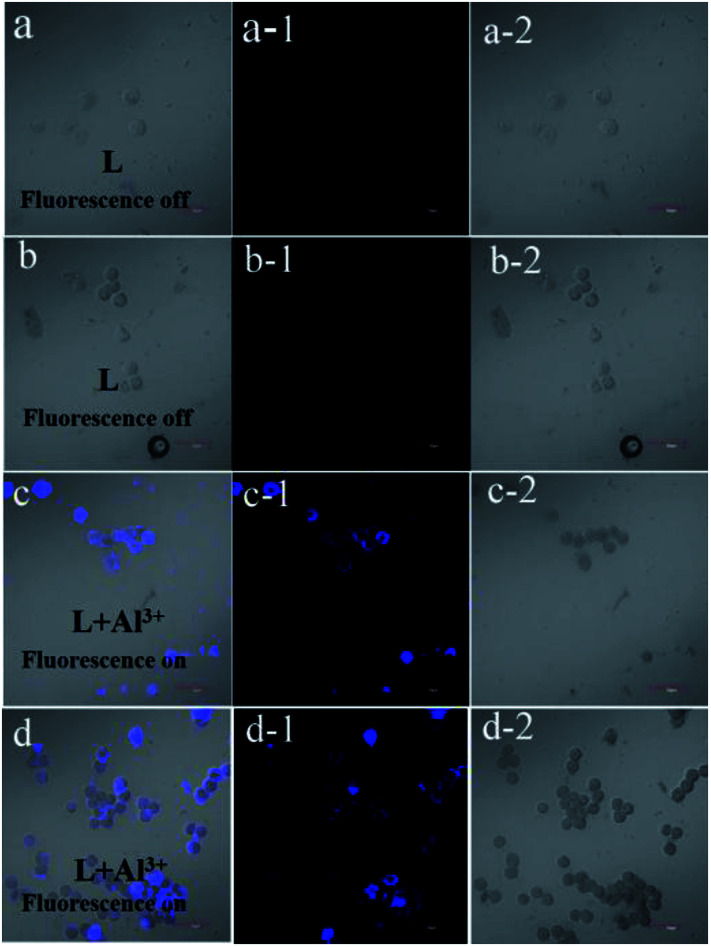
The fluorescence images of HeLa cells before and after addition of Al^3+^ (1 × 10^−5^ M) in the solution of L (1 × 10^−5^ M). The image of the cell containing L ((a)–(a-2) and (b)–(b-2)), the cell image of L after the addition of Al^3+^ ((c)–(c-2) and (d)–(d-2)).

**Fig. 8:**
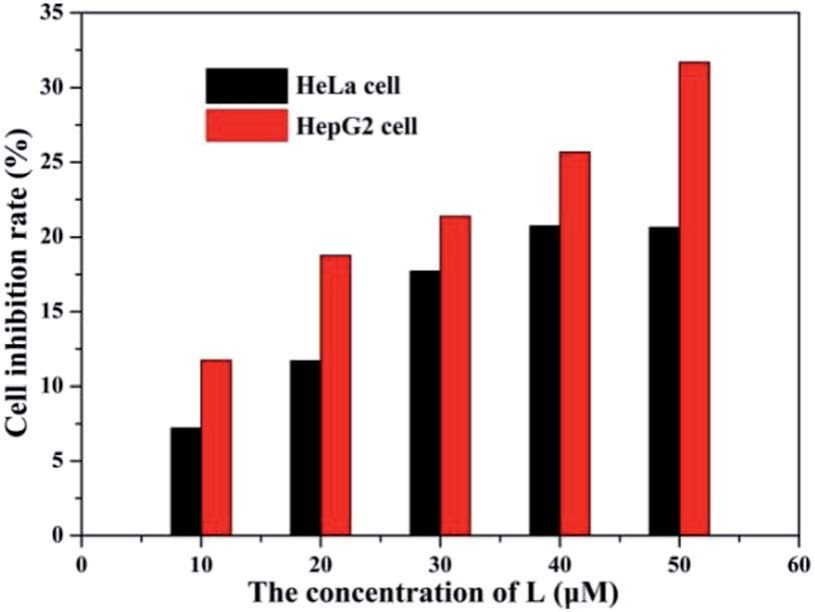
The cytotoxicity of L for the HeLa and HepG2 cells at various concentration (10, 20, 30, 40, and 50 μM).

**Fig. 9:**
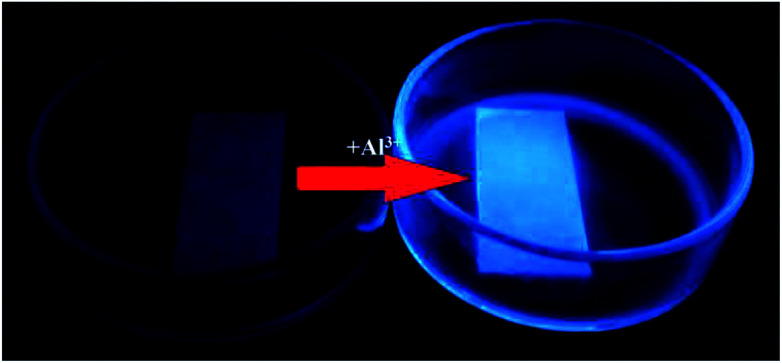
The fluorescence change of the filter paper in the process of sensing Al^3+^ in C_2_H_5_OH/H_2_O media.

The fluorescence microscopy images of HeLa cells with L and L–Al^3+^ demonstrated the sensor's ability for sensing Al^3+^ in biological systems. The HeLa cells were treated with L for 30 min and the fluorescence signal was recorded. The HeLa cells showed no fluorescence signals. Then, the aqueous solution of Al^3+^ was added to the HeLa cells treated with a solution of L. As shown in Fig. 7, the HeLa cells showed intense blue fluorescence, and the cells were also labelled clearly. The results suggested that the sensor could permeate the plasma membrane of the HeLa cells and give specific blue fluorescence signal in the presence of Al^3+^. The experiment demonstrated that L could act as a highly selective fluorescence sensor for Al^3+^ in biological systems. Moreover, the cytotoxicity (HeLa cells and HepG2 cells) of L was tested by CCK8 methods. As shown in Fig. 8, with increasing concentration (10 μM, 20 μM, 30 μM, 40 μM, and 50 μM) of L, the inhibition rate was lower for the HeLa cells compared with the HepG2 cells. Under the condition of high concentration (50 μM), the inhibition rate for the HepG2 cells was still lower than 35%. This showed that the sensor was suitable for use for a variety of biological applications. In addition, simple paper chromatography was performed to evaluate the application of the sensor in environmental samples. As shown in Fig. 9, the paper chromatogram soaked by the sensor solution could detect Al^3+^ in a low concentration aluminium ion solution.

## Experimental

### Materials

All the chemicals containing solvents were of reagent grade and were used without further purification. The HeLa cells were provided by the Bioeagle Technology Limited Corporation, Wuhan, P. R. China.

### Synthesis and characterization of the nitrogen heterocycle (L)

**Scheme 1:**
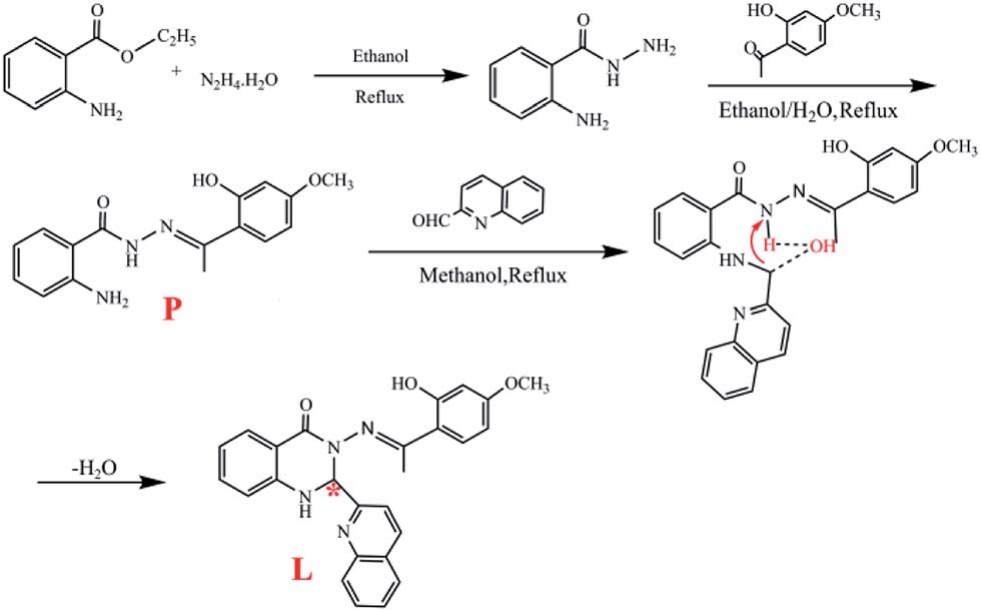
The synthetic route of the target molecule L (the asterisk is for the chiral carbon).

The synthetic routine is shown in Scheme 1.

The precursor (P) of L was synthesized and characterized by the reported method and was used without further characterization.^28^

**Fig. 10:**
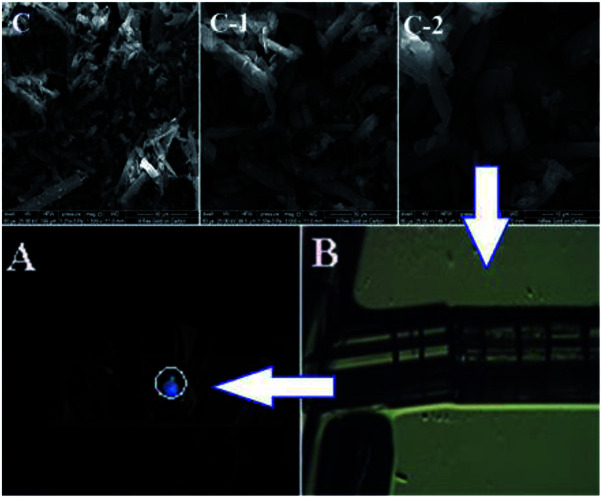
The SEM images (C, C-1, and C-2) and fluorescence microscopy images (A and B) of the crystal of L.

A methanol solution (5.0 mL) of quinoline-2-carbaldehyde (0.1 mol, 0.157 g) was added to another methanol solution (10 mL) containing P (0.1 mol, 0.327 g). Then, the solution was refluxed for 10 h and cooled to room temperature, which lead to the appearance of a white precipitate. The mixture was filtered and dried under vacuum. Recrystallization from CH_3_OH/H_2_O (v : v = 1 : 2) gave the target product L which was dried under vacuum. Yield: 75%, mp: 260–261 °C. ^1^H NMR (DMSO–d_6_ 400 MHz, Fig. 5): *δ* 12.52 (1H, s, –O^18^–H), *δ* 8.10–8.12 (1H, d, –C^9^–H), *δ* 7.93–7.99 (2H, m, –C^5,6^–H), *δ* 7.87 (1H, s, –N^8^–H), *δ* 7.75–7.78 (2H, m, –C^4,7^–H), *δ* 7.67–7.69 (1H, d, –C^1^–H), *δ* 7.60–7.63 (1H, m, –C^13^–H), *δ* 7.56–7.58 (1H, d, –C^10^–H), *δ* 7.30–7.33 (1H, m, –C^14^–H), *δ* 6.75–6.79 (2H, m, –C^2,3^–H), *δ* 6.44–6.46 (1H, d, –C^11^–H), *δ* 6.27–6.31 (2H, d, –O^12,15^–H), *δ* 3.71 (3H, s, –C^16^–H), *δ* 2.31 (3H, s, –O^17^–H). ^13^C NMR (DMSO–d_6_ 400 MHz, Fig. S1†): 174.06, 163.24, 161.90, 160.76, 158.94, 147.03, 137.93, 134.43, 131.54, 130.50, 120.42, 128.38, 128.03, 127.52, 119.48, 118.25, 114.99, 114.35, 112.14, 106.68, 101.64, 76.33, 55.77, 17.45. In the compound, there is a chiral carbon atom (Scheme 1, asterisk). HRESI-MS for C_26_H_22_O_3_N_4_: 439.1749 (molecular ion peak). The solid powder of L exhibited a relatively regular columnar configuration as seen in the SEM images (Fig. 10(C)–(C-2)).

Moreover, the X-ray diffraction measurement for L (size: 0.40 mm × 0.10 mm × 0.10 mm) was performed on a Bruker SMART APEX II CCD diffractometer equipped with a graphite monochromatized Mo Kα radiation (*λ* = 0.71073 Å) by using φ–ω scan mode. Semi-empirical absorption correction was applied to the intensity data using the SADABS program. The structure was solved by direct methods and refined by full matrix least-square on *F*^2^ using the SHELXTL-97 program. All non-hydrogen atoms were refined anisotropically. All H atoms were positioned geometrically and refined using a riding model. Details of the crystal parameters, data collection, and refinements for L are summarized in Table 1.

**Table 1:** Crystal data and structure refinement for compound L

	L
Empirical formula	C_26_H_22_N_4_O_3_
Formula weight	438.48
*T*/K	296
Crystal system	Triclinic
Space group	*P*1̄
*a*/nm	6.4124(14)
*b*/nm	12.451(3)
*c*/nm	14.248(3)
*α*/(°)	101.099(4)
*β*/(°)	102.349(3)
*γ*/(°)	94.856(3)
*V*/nm^3^	1.0810(4)
*Z*	2
*D*_c_ (g cm^−3^)	1.344
Absorption coefficient/mm^−1^	0.090
*F* (000)	458.0
Reflection collected, unique (*R*_int_)	3780, 2049
Data, restraint, parameter	2049, 0, 302
Goodness of-fit (GOF) on *F*^2^	0.997
Residuals *R*_1_, w*R*_2_	0.0563, 0.1514

**Fig. 11:**
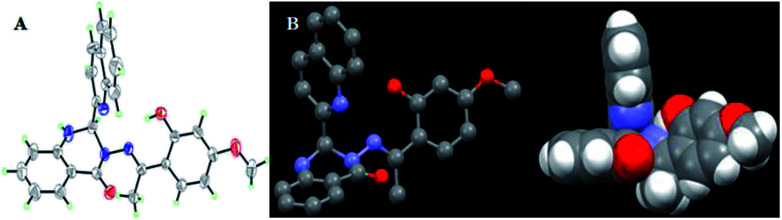
The ORTEP drawing of L with 30% thermal ellipsoids (A); the molecular crystal structure of L and H-atoms are omitted for clarity (B).

Single crystal X-ray structure (Fig. 11 A, ORTEP drawing) analysis of L reveals that it crystallizes in the triclinic *P*1̄ space group. The crystal structure of L is in accordance with the structure from NMR and HRESI-MS, which exhibited distorted non-planar configurations. In solution, L showed no fluorescence, however, its solid crystal (Fig. 10 B) showed blue fluorescence (Fig. 10 A). The distorted rotation (Fig. 11 B) of the quinoline ring and paeonol in solution was deduced to give rise to the fluorescence quenching.

**Fig. 12:**
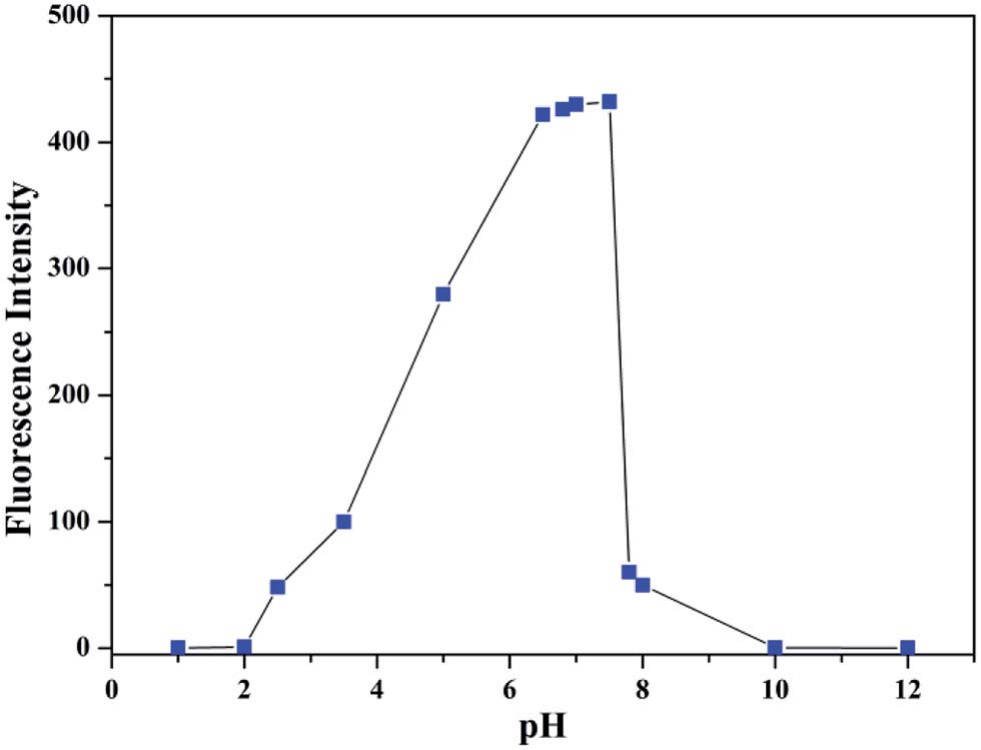
The fluorescence intensity of L at various pH values in the presence of Al^3+^.

All spectroscopic measurements were performed in C_2_H_5_OH/H_2_O (4 : 1) solution. To evaluate the influence of pH on the fluorescence, the fluorescence intensity of L at various pH values in the presence of Al^3+^ was tested (Fig. 12). Under strongly acidic conditions (<2.0) and under alkaline conditions (>8.0), the fluorescence of Al^3+^–L disappeared. In the range of 6.5 to 7.5, the fluorescence exhibited was stronger. Therefore, by the fluorescence screening experiment, pH = 7.2 was chosen as the appropriate testing pH. In addition, the neutral pH is in concurrence with the pH in physiological conditions. The pH (pH = 7.2) of the solution was controlled by the HEPES buffer solution. Stock solutions (1.0 × 10^−3^ M) of metal ions (metal nitrate) were prepared in double-distilled water. The stock solution of 1.0 × 10^−3^ M was prepared in C_2_H_5_OH/H_2_O (4 : 1) and the pH was controlled by the HEPES buffer solution (pH = 7.2). In the titration experiments, each time 2 mL aqueous solution containing 10 μL solution of L (1.0 × 10^−3^ M) was filled in a quartz optical cell of 1 cm optical path length. Then, 10 μL of Al^3+^ stock solution was added to the compound solution with a micro-pipette. Spectral data were recorded 0.5 min after the addition. In the selectivity experiment, the test samples were prepared by mixing appropriate amounts of the metal ion stock solution into 2 mL C_2_H_5_OH/H_2_O solution of L (10 μL). For fluorescence measurements, excitation wavelength was at 350 nm.

The binding constant for L–Al^3+^ was calculated by the linear Benesi–Hildebrand expression.^29,30^
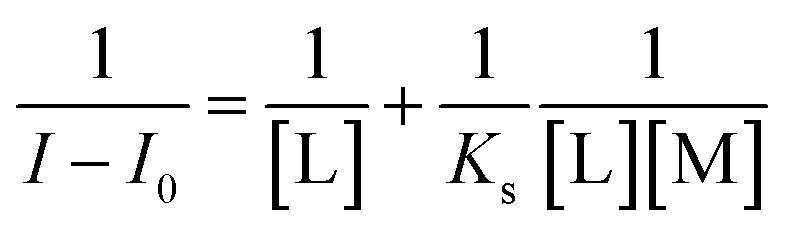
where, *I* is the change in fluorescence intensity in the presence of Al^3+^ at 451 nm, *K*_s_ is the stability constant, and [L] and [M] is the concentration of L and Al^3+^, respectively. *I*_0_ is the fluorescence intensity of L in the absence of Al^3+^. On the basis of the plot of 1/(*I* − *I*_0_) *versus* 1/[Al^3+^], the stability constant can be obtained.

### Physical measurement

NMR spectra were recorded on a Varian VR400-MHz spectrometer with TMS as the internal standard. The melting points of the compounds were determined on a Beijing XT4-100X microscopic melting point apparatus. The UV-Vis spectra were recorded on a Perkin-Elmer Lambda-35 UV-Vis spectrophotometer. Fluorescence spectra were obtained on a Cary Eclipse spectrophotometer at room temperature. The fluorescence image of the HeLa cells was obtained using Olympus FV1300 laser confocal fluorescence microscope. The crystal data were collected by the Bruker Smart-Apex-II instrument. The SEM images were obtained by F250 thermal field emission scanning electron microscope.

### Cell imaging and cytotoxicity

The HeLa cells were cultured in a flask in Dulbecco's modified Eagle's medium supplemented with a heat-inactivated bovine serum (10%), 100 U mL^−1^ penicillin, and 100 U mL^−1^ streptomycin and were maintained at 37 °C in a humidified atmosphere (5% CO_2_ and 95% air). The cells were seeded in a six orifice plate and allowed to adhere for 12 h before treatment. Then, the cells were incubated with fresh media containing 100 μL of the sensor solution (dissolved in DMSO/C_2_H_5_OH (1 : 4), 1 mM). After 0.5 h, the growth medium was removed, and the cells were washed with saline solution several times. The cover glass was then mounted on a microscope glass slide and was studied under a microscope. Then, 200 μL fresh water media containing Al(NO_3_)_3_ (dissolved in H_2_O, 1 mM) was added to the above cell solution containing L. After the cells were incubated for 0.5 h, they were washed with saline solution several times, and were then studied under a microscope. Imaging was performed using an Olympus FV1300 laser confocal fluorescence microscope (excitation wavelength: 355 ± 10 nm).

The cytotoxicity of L towards the HeLa and HepG2 cells was evaluated by the following procedure. The cultured cells were placed in a sterilized phosphate buffer solution. After that, the cells were treated with the L solution (DMSO solvent) at various concentrations (10, 20, 30, 40, and 50 μM). After incubation for 12 h, 100 μL of CCK8 culture medium solution was added to the above solution, and incubated for 0.5 h. The absorption was measured at 450 nm. The cytotoxicity of L was evaluated by measuring the cell viability, which was achieved with a hemacytometer under a microscope. The cytotoxicity of the solvent was deducted.

## Conclusions

In summary, we have fabricated a highly selective fluorescence sensor for Al^3+^ derived from a nitrogen heterocyclic compound. The fluorescence response mechanism due to the synergistic effect of photoinduced electron transfer (PET) and C=N isomerization was concluded by the fluorescence and energy change processes accompanying the no-fluorescence to intense blue fluorescence states. By structure analysis, it was found to exhibit the unusual double ion coordination configuration. Moreover, the sensor showed high sensitivity for sensing Al^3+^ in C_2_H_5_OH/H_2_O media. In addition, the cell fluorescence imaging was also investigated with HeLa cells, which exhibited high penetration depth. The coordination sites on the sensor L for binding Al^3+^ were confirmed by NMR and MS titrations. Such investigations endowed the potential application of sensor for tracking and detecting of Al^3+^ in biological and environment fields.

## Conflicts of interest

There are no conflicts to declare.

## Supplementary Material

RA-009-C8RA10036E-s001

RA-009-C8RA10036E-s002
