# Supplementary material for: A novel nitrogen heterocycle platform-based highly selective and sensitive fluorescence chemosensor for the detection of Al3+ and its application in cell imaging
Source: RSC Adv. 2019 Feb 12;9(10):5377–83. doi: 10.1039/c8ra10036e (PMC9060657; doi:10.1039/c8ra10036e)
Supplement: RA-009-C8RA10036E-s001 [file RA-009-C8RA10036E-s001.pdf]

Electronic Supplementary Material (ESI) for RSC Advances.
This journal is © The Royal Society of Chemistry 2019

A novel nitrogen heterocyclic platform based highly selective and sensitive fluorescence chemosensor for the detection of Al^{3+} and its application in cell imaging

Zengchen Liu, Shujing Li, Genwu Ge, Yanxia Li, Chunxiang Zhao, Hui Zhang,* Zhiguang Yang*

College of Chemistry and Chemical Eningeering; Henan Key Laboratory of Rare Earth Functional Materials; International Joint Research Laboratory for Biomedical Nanomaterials of Henan; The Key Laboratory of Rare Earth Functional Materialsi and Applications, Zhoukou Normal University, Zhoukou 466001, P.R. China

*Corresponding author. Tel.: +86 0394 8718252; Fax: +86 0394 8178252.

E-mail address: liuzengchen@zknu.cn

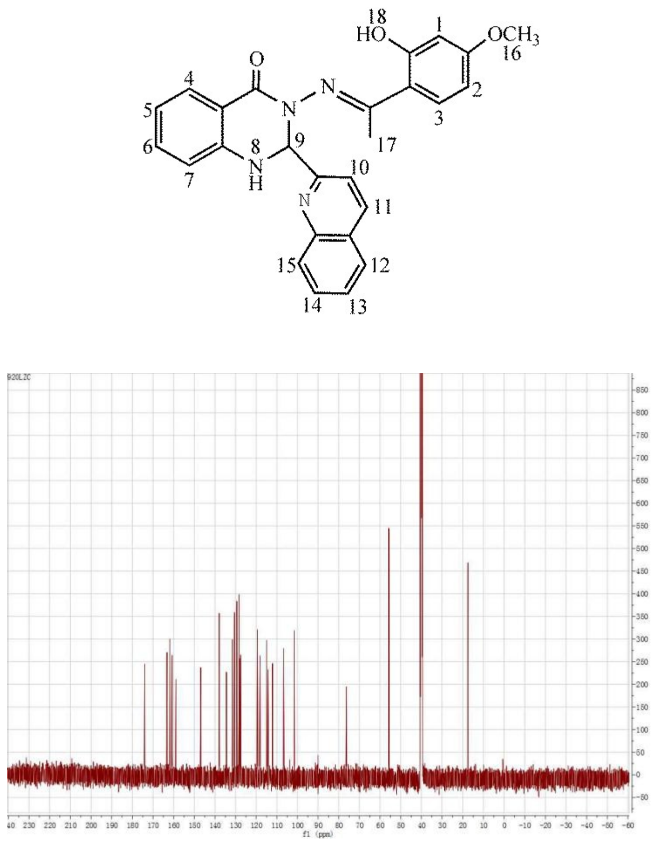

Fig. S1 The ^{13}CNMR spectrum of L
